# First reported case of human Camelpox in Qatar: A case report

**DOI:** 10.1016/j.idcr.2026.e02518

**Published:** 2026-02-08

**Authors:** Hani Hamad, Junais Koleri, Jabeed Parengal, Alaaeldin Abdulmajed, Mohamed Ali Ben Hadj Kacem, Manal Hamed, Peter Coyle, Muna Al Maslamani

**Affiliations:** a Department of Infectious Diseases, Communicable Diseases Centre, Hamad Medical Corporation, Doha, Qatar; b Department of Microbiology and Pathology, Hamad Medical Corporation, Doha, Qatar

**Keywords:** Camelpox, Orthopoxvirus, Zoonotic infections

## Abstract

Camelpox is a zoonotic viral infection caused by the *Camelpox virus* (CMLV), a member of the *Orthopoxvirus* genus. The disease is endemic in camel-rearing regions of Africa, the Middle East, and Asia. Human infection is rare and typically occurs following direct contact with infected camels or their lesions. We report the first documented case of human camelpox in Qatar. The diagnosis was established based on epidemiological linkage to infected camels, compatible clinical presentation, and confirmatory laboratory testing.

## Introduction

Camelpox is a viral disease caused by *Camelpox virus* (*CMLV*), a member of the *Orthopoxvirus* genus within the *Poxviridae* family [1]. The genome of camelpox virus consists of a single linear double-stranded DNA molecule, organized into densely packed coding regions and replicating entirely within the host cell cytoplasm. The complete genome measures approximately 205.7 kilobase pairs [2].

The disease is en-zootic in dromedary camels (*Camelus dromedarius*) across Africa, the Middle East, and Asia, where outbreaks have been reported in camel-rearing regions [3]. While camelpox is primarily an animal disease, sporadic human infections have been documented, typically among individuals with direct exposure to infected camels [4].

Clinical manifestations in humans are typically limited to localized cutaneous lesions and are characterized by painful papulopustular skin lesions. It may also lead to mucocutaneous ulcers on the lips and mouth, particularly in immune-compromised individuals. Mild systemic symptoms such as fever and malaise may also occur [5], [6].

Unlike *Monkey pox virus* (*MPXV*), which can spread through human-to-human transmission, *CMLV* is considered a zoonotic infection with no evidence of sustained human transmission [4]. The primary mode of transmission to humans is through direct contact with infected camels, particularly during handling, milking, slaughtering, or veterinary care [7].

Despite the potential public health significance of *CMLV*, human cases remain underreported due to limited diagnostic capabilities and a lack of awareness among healthcare providers. The clinical overlap between camelpox, monkeypox, and other *Orthopoxvirus* infections further complicates diagnosis. Laboratory confirmation relies on polymerase chain reaction (PCR) testing for *Orthopoxvirus*, as specific *CMLV* PCR assays are not widely available [8].

According to national livestock statistics, Qatar’s camel population has shown a steady increase over the past five years. Ministry of Municipality records indicate a rise from 105,387 camels in 2017–150,565 camels in 2021, reflecting an average annual growth rate of 9 % [9]. An outbreak occurred in the United Arab Emirates in the summer of 2020, producing severe external and internal lesions in affected animals [10]. However, there are no published reports of similar herd-level outbreaks in Qatar. Additionally, there was no reported human *CMLV* in Qatar or the Gulf region.

In this case report, we describe the first documented case of human camelpox in Qatar. It highlights the zoonotic potential of *CMLV* and the importance of occupational exposure in viral transmission. It also highlights the need for increased surveillance, improved diagnostic tools, and preventive measures among individuals working closely with camels.

## Case presentation

A 30-year-old previously healthy male from Bangladesh presented to a primary healthcare center with painful pustular lesions on his arms of 5 days duration. The lesions are painful and mildly pruritic. They were located as one lesion on his right forearm and two similar lesions on his left arm. He had no mucosal lesions. He reported subjective fever and malaise a day prior to presentation. He worked on a camel farm performing animal care, grooming, milking, and assisting with births. He helped with the delivery of a newborn camel that later developed similar lesions. He frequently washed the animal with bare hands. He denied sexual activity or close contact with sick patients. The patient had resided in Qatar for the past ten years, last traveled to Bangladesh three years prior.

**Fig. 1:**
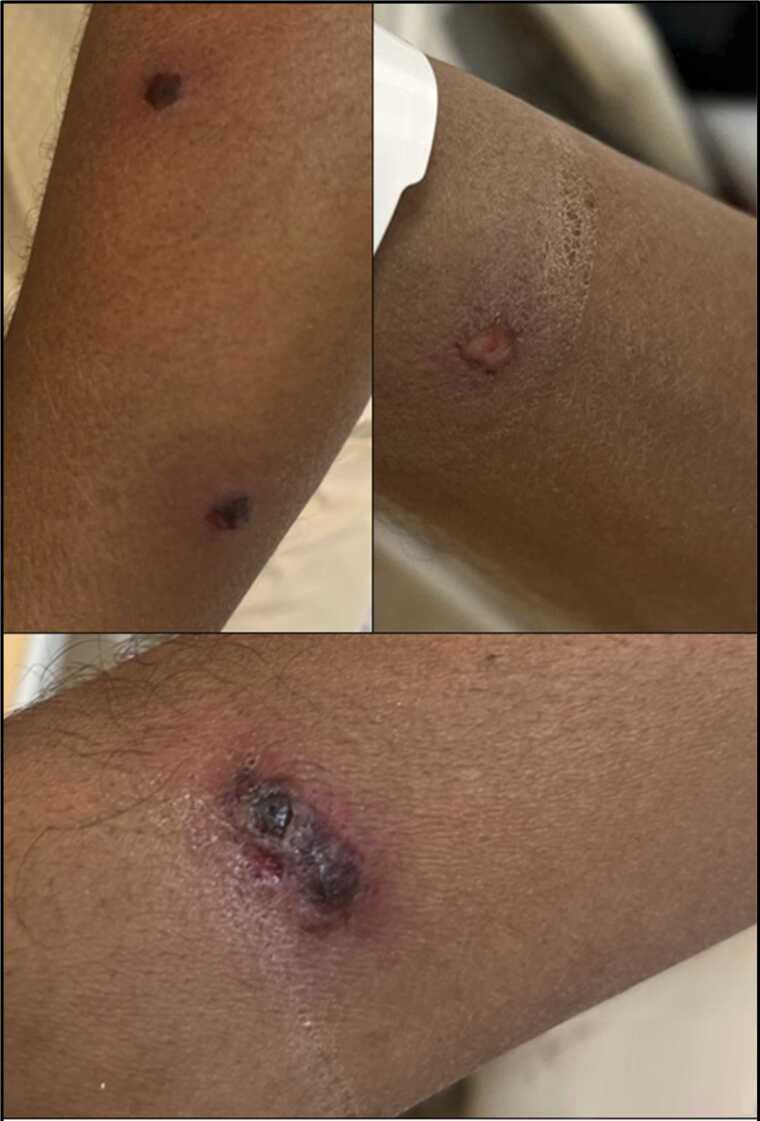
Ulcerated necrotic skin lesion on the forearm showing a central black eschar with surrounding erythema and induration, characteristic of the cutaneous manifestation of camelpox infection.

Upon examination, the patient appeared in no distress and was vitally stable. Examination of the lesions revealed well-demarcated ulcerated lesions on both forearms, the largest was approximately 1.5–2 cm in diameter. One of the lesions had a central necrotic eschar with a dark crusted surface, surrounded by an erythematous and slightly indurated border. The adjacent skin appears mildly edematous, with no evidence of active purulent discharge **(**Fig. 1**)**. There is no visible lymphangitic streaking. The rest of the examination was unremarkable with no lymphadenopathy or mucosal lesions.

A swab sample from one lesion tested positive for *Orthopoxvirus* via polymerase chain reaction (PCR). Given concerns for possible *Camelpox virus* or *Mpox virus* (*MPXV*) infection, the Infectious Diseases (ID) team recommended further evaluation and isolation.

Swab specimens collected from skin lesions on the forearms were transported in a Universal Transport Medium (UTM) and sent for analysis. Nucleic acid extraction was done using the EZ1&2 Virus Mini Kit v2 on the EZ1® Advanced XL instrument. Then, screening for Orthopoxvirus (non-variola) species was done using the RealStar® Zoonotic Orthopoxvirus PCR Kit 1.0 based on real-time PCR technology. Confirmation and identification of the Mpox virus was attempted using the UK Health Security Agency in-house real time PCR protocol. Camelpox detection and identification was done using the real time PCR Primerdesign Ltd Camelpox virus genesig Standard Kit. All real time PCR test were performed on the Applied Biosystems QuantStudio™ 5 Real-Time PCR System.

The sample was positive for Orthopoxvirus screening showing a Ct value of 19.62. Mpox real time PCR showed negative results and Camelpox real time PCR showed positive results with a Ct value of 17.39.

Given the epidemiological link and the unavailability of a specific *CMLV* PCR test, the diagnosis of human camelpox was strongly supported based on clinical presentation and *Orthopoxvirus* PCR positivity.

## Discussion

We present the first case of documented human *CMLV* in Qatar. The diagnosis was based on the history of exposure, clinical presentation and was confirmed by serological testing. This case illustrates the relevance of the One Health approach in understanding and managing zoonotic infections arising at the human–animal–environment interface.

This case underscores the importance of occupational exposure to zoonotic infections. The patient’s direct handling of an infected camel without protective gloves likely facilitated viral transmission. Previous reports indicate that human cases of camelpox are self-limiting, with lesions resolving within a few weeks without significant complications [5]. However, severe cases have been documented in immunocompromised individuals [7]. Fortunately. Our patient has a benign and smooth clinical course with no systemic complications.

From a One Health perspective, effective prevention of camelpox requires coordinated surveillance in both human and animal populations. Routine veterinary monitoring of herds, timely reporting of outbreaks, and molecular surveillance of circulating viral strains are essential for early detection. Environmental factors, including farm hygiene, animal housing conditions, and waste management practices, may further influence viral persistence and transmission dynamics [11].

In Qatar, where camel farming and racing are widespread, this case emphasizes the need for integrated occupational health programs targeting camel workers. These programs should include health education, provision of personal protective equipment, routine screening of animals, and standardized reporting pathways between veterinary and healthcare systems. Strengthening collaboration between public health authorities, veterinary services, and environmental agencies is critical for outbreak preparedness.

Given the endemic nature of *CMLV* in camels and the occupational risk among camel workers, preventive measures are crucial. The use of protective gloves, improved hygiene practices, and awareness among at-risk populations can help reduce transmission [4]. Enhanced surveillance and diagnostic capabilities are also necessary, particularly in regions where camels play a significant role in agriculture and trade.

Efforts against poxvirus and biological safety measures in the Middle East have been reviewed. The measures included public education campaigns, good hygiene and sanitation promotion, and strict infection control measures in healthcare and veterinary settings [12]. Research on CMLV-based vaccines has shown that they can effectively protect against camelpox in animals. The Jordanian Vaccine Company (JOVAC) developed the Orthovac-R vaccine, and similar vaccines have been used in Egypt, Morocco, and Russia. In a study of 101 farms, comparing vaccinated and unvaccinated animals, vaccination proved beneficial during a natural lumpy skin disease outbreak [13]. This evidence supports Jordan’s continued use of the vaccine as an important control measure. Widespread implementation of vaccination programs may reduce viral circulation in animal reservoirs and subsequently lower zoonotic risk.

Overall, this case highlights the importance of integrating clinical medicine, veterinary public health, and environmental management to mitigate zoonotic threats and strengthen regional health security.

## Conclusion

We report the first documented case of human camelpox in Qatar. This case highlights the zoonotic potential of *Camelpox virus* and the occupational risks faced by camel workers. Importantly, it emphasizes the value of a One Health approach that integrates human, animal, and environmental health sectors. It also underscores the need for increased surveillance, preventive measures, and improved diagnostic capabilities to mitigate further transmission.

## Ethics approval and consent to participate

Institutional ethics approval was not required for this case report and a waiver was obtained.

## Author agreement

None.

## Originality & Exclusivity

This manuscript is original, has not been published previously, and is not under consideration elsewhere.

## Authorship & Approval

− All authors listed have made substantial contributions to the work.
− All authors have read and approved the final version of the manuscript and agree to its submission.
− The order of authorship has been approved by all authors.

## Author statement

HH and JK were responsible for patient management and data collection. HH wrote the original draft. JP and AA contributed to clinical investigation and interpretation of findings. MK, MH, and PC performed laboratory analysis and diagnostic confirmation. MM supervised the study and provided critical intellectual input. All authors contributed to manuscript drafting, revision, and approved the final version.

## CRediT authorship contribution statement

**Manal Hamed:** Methodology, Investigation. **Peter Coyle:** Methodology, Investigation. **Alaaeldin Abdulmajed:** Supervision, Conceptualization. **Mohamed Ali Ben Hadj Kacem:** Methodology, Investigation. **Muna Al Maslamani:** Validation, Supervision, Conceptualization. **Junais Koleri:** Writing – review & editing, Data curation, Conceptualization. **Jabeed Parengal:** Writing – review & editing, Data curation, Conceptualization. **Hamad Hani Ghassan:** Writing – review & editing, Writing – original draft, Data curation, Conceptualization.

## Declaration of Competing Interest

The authors declare no conflicts of interest.
